# A validation study of the Multidimensional Life Satisfaction Scale for Children

**DOI:** 10.1186/s41155-017-0068-6

**Published:** 2017-07-06

**Authors:** Cynthia Cassoni, Edna Maria Marturano, Susana Coimbra, Anne Marie Fontaine

**Affiliations:** 1 Psychology Department, FFCLR, University of São Paulo, Av. Bandeirantes, 3900, Bairro Monte Alegre, CEP 14040-901 Ribeirão Preto, SP Brazil; 2 Faculty of Psychology and Educational Sciences of the University of Porto, Porto, Portugal; 3 Ribeirão Preto Medical School, University of São Paulo, Av. Bandeirantes, 3900, Bairro Monte Alegre, CEP 14040-901 Ribeirão Preto, SP Brazil; 4 Center for Psychology at University of Porto, Faculty of Psychology and Education Sciences, University of Porto, Rua Alfredo Allen, Porto, 4200-135 Portugal

**Keywords:** Instrument validation, Life satisfaction, Confirmatory factor analysis

## Abstract

**Introduction:**

Recent studies on the life satisfaction in children and young people have investigated its association with vulnerability, discrimination, the individual’s school environment and network of relationships, and mental health. The growing interest in the area demands instruments with good psychometric properties.

**Aim:**

The aim of this study is to study the psychometric properties of the Multidimensional Life Satisfaction Scale for Children (MLSS-C).

**Method:**

The participants were 379 elementary schoolchildren aged 9 to 14 (*M* = 10.5 years), enrolled in public schools in a city in the state of São Paulo, Brazil. The instruments used were the MLSS-C, the Self-Description Assessment Questionnaire 1 (SDQ1), the Social Skills Rating System (SSRS), and the Childhood Stress Scale (CSS). Two data collections were made, one in the fifth year of elementary school and one in the sixth. Confirmatory factor analysis (CFA) was carried out to assess the structural model’s goodness of fit. The internal consistency (Cronbach’s alpha and composite reliability), test-retest reliability and the discriminant, convergent, and divergent validity were also assessed.

**Results:**

Regarding CFA, after removing items with saturation values below .50, six dimensions proposed by the authors remained, five of them with alpha values above .70. The construct validity was confirmed by finding moderate and positive correlations between life satisfaction and self-concept and social skills (convergent validity) and lower and negative correlation with childhood stress (divergent validity).

**Conclusion:**

Together, the reported results provide preliminary evidence of the reliability and validity of this scale. It is suggested, therefore, that this scale is suitable for both research and practice with Brazilian schoolchildren.

## Background

Studies in the area of psychology have turned to examining the role of life satisfaction in the adjustment of people in life stages other than adulthood, as a reflection of society and the changes it has experienced. Research that initially focused on adults (e.g., Solomon & Winfield, 1945) was extended to adolescents and children in the 1970s and 1980s (see Daly & Carpenter, 1985; Lessing, 1972). A search made by the authors in the PsycNET database indicates that more than 6000 articles on life satisfaction have been published up to 2016, but only a quarter of them focus on adolescents (13–17 years) and schoolchildren (6–12 years).

Satisfaction with life can be defined as the overall assessment of quality of life made by people according to their own criteria (Diener, Suh, Lucas, & Smith, 1999; Huebner, 1994) or as a cognitive response referring to evaluative judgments of satisfaction with life as a whole or in its multiple dimensions (Lucas, Diener, & Suh, 1996). This definition somehow implies the cognitive and emotional ability to consider and weigh several different sources of information.

For Lucas, Diener, and Suh (1996), satisfaction with life is (along with positive and negative affect) one of the dimensions of subjective well-being that refers to the frequency of positive or negative feelings. In other studies, however, the concepts of satisfaction with life and subjective or general well-being are usually superimposed or interchangeable (Cheung & Lucas, 2015; Kuppens, Realo, & Diener, 2008; Lyubomirsky, King, & Diener, 2005; Pavot & Diener, 2008; Poletto & Koller, 2011).

Studies on school-age children and adolescents have found associations between satisfaction with life and vulnerability and discrimination (Gilligan & Huebner, 2002; Poletto & Koller, 2011), school environment (Gilligan & Huebner, 2002; Poletto & Koller, 2011), work (Arteche & Bandeira, 2003), and an individual’s network of relationships (Serafini & Bandeira, 2011). Satisfaction with life has also been associated with perceptions of support from teachers (Guess & McCane-Bowling, 2016) and parents (Jiang, Huebner, & Hills, 2013), as well as satisfaction with the neighborhood (Shin, Morgan, Buhin, Truitt, & Vera, 2010). Satisfaction with life also predicts externalizing and internalizing problems (Gao et al., 2015; Haranin, Huebner, & Suldo, 2007).

Given the multiplicity and diversity of these associations, researchers have proposed models that cover the unidimensionality and multidimensionality of life satisfaction. The unidimensional, general, or global models (Diener, Emmons, Larsen, & Griffin, 1985; Pavot & Diener, 1993; Huebner, 2004) use a single scale to indicate levels of satisfaction with life in general. The multidimensional models (Giacomoni & Hutz, 2008; Huebner, 1994) provide a profile of satisfaction with life in various specific domains.

Proctor, Linley, and Maltby (2009) have reviewed the instruments developed from the unidimensional and multidimensional models to assess life satisfaction. Among the models of the second group, the authors present the Multidimensional Students’ Life Satisfaction Scale (MSLSS) (Huebner, 1994), a 40-item self-report scale designed to provide a profile of life satisfaction within five specific domains (family, friends, school, context, and self) and an overall assessment of general life satisfaction. The MSLSS is applicable for use with students aged 8–18. High indices of internal consistency were reported for each dimension (.82 for family, .85 for school, .85 for friends, .83 for context, and .82 for self) and for the full scale (.92).

Today, the MSLSS is used in different cultures, having been adapted and validated for young people in France (Fenouillet, Heutte, Martin-Krumm, & Boniwell, 2015), Iran (Hatami, Motamed, & Ashrafzadeh, 2010), Turkey (Irmak & Kuruüzüm, 2009), Serbia (Jovanovic & Zuljevic, 2013), and Italy (Zappulla, Pace, Cascio, Guzzo, & Huebner, 2014). The studies of adaptation for these countries clearly presented the stages of translation and semantic adaptation, exploratory and confirmatory factor analysis (CFA), and reliability indices of the instrument, maintaining the five-dimension structure, with good indices of fit and satisfactory internal consistency values (Cronbach’s alpha greater than .70).

The studies also explored the construct validity of the scale. For convergent validity, instruments were used to measure self-esteem (Jovanovic & Zuljevic, 2013; Zappulla et al., 2014), self-concept (Zappulla et al., 2014), positive relationships with others (Zappulla et al., 2014), positive affect (Jovanovic & Zuljevic, 2013), and other instruments of life satisfaction (Fenouillet et al., 2015; Irmak & Kuruüzüm, 2009). For divergent validity, instruments were used to measure depression (Fenouillet et al., 2015; Jovanovic & Zuljevic, 2013; Zappulla et al., 2014), mental health (Zappulla et al., 2014), and anxiety and stress (Jovanovic & Zuljevic, 2013).

Giacomoni and Hutz (2008) developed the Multidimensional Life Satisfaction Scale for Children (MLSS-C), a Brazilian instrument, based on the scale originally developed by Huebner (1994), seeking to adapt Huebner multidimensional model to the characteristics of the Brazilian culture. To ensure the ecological validity of the scale, the authors derived the MLSS-C items mainly from interviews conducted with Brazilian children aged 5–12.

The MLSS-C contains 50 items, distributed among six dimensions, including domains of life satisfaction not covered in Huebner’s original model, such as compared-self and non-violence. The *self* dimension consists of items that describe the self with positive characteristics, such as self-esteem, good mood, the ability to relate, and the ability to show affection. The *compared-self* dimension groups items characterized by evaluations comparative with peers. The *non-violence* dimension includes items with content associated with aggressive behaviors. The *family* dimension includes items that describe a healthy, harmonious, and affectionate family environment and satisfying relationships. Items in the *friendship* dimension refer to relationships with peers, the level of satisfaction in these relationships, and some indications of leisure, fun, and support. Finally, the *school* dimension includes items that describe the importance of school, the school environment, interpersonal relationships in this space, and levels of satisfaction with this environment.

Giacomoni and Hutz (2008) investigated the psychometric properties of the MLSS-C. They first conducted an exploratory factor analysis (EFA) with a sample of 661 students aged 7–12 (mean 10.6) from public and private schools in Porto Alegre in Rio Grande do Sul. The EFA provided a six-factor structure as the best solution, with factors corresponding to the proposed model. Five dimensions presented internal consistency rates ranging from .82 and .86; only one dimension, non-violence, obtained an index of less than .70 (.66). The total Cronbach’s alpha was .93. In a further study with 230 children, the authors found moderate positive correlations between all dimensions and a measure of self-esteem, as well as moderate negative correlations with measures of depression and anxiety (Giacomoni & Hutz, 2008).

The current evidence of adequate psychometric properties of the MLSS-C indicates that it is worth pursuing the validation process. As part of this process, there is a need to confirm the factorial structure derived from the EFA. Will the six-factor model maintain in a CFA? Given Brazil’s cultural diversity, will data from children in another region fit the same model? These questions guided the present study, which aims to validate the structure of the scale using CFA in a sample of children living in the state of São Paulo, Brazil. The sample participated in a broader prospective study from the fifth to the sixth year of elementary school.

In addition to the factorial validity, other contributions to the construct validity are presented through the study of some indicators of discriminant, convergent, and divergent validity. For the discriminant validity, each dimension’s average variance extracted (AVE) must be compared to its squared correlations with other dimensions in the model (Fornell & Larcker, 1981).

Convergent validity is demonstrated when there are correlations between instruments that measure similar traits or characteristics (Pasquali, 2007). Social skills and self-concept constructs were therefore chosen which (according to the literature) are moderately and positively associated with self, family, school and friendship dimensions of life satisfaction as measured by the MSLSS, an instrument similar to the MLSS-C (Jovanovic & Zuljevic, 2013; Zappulla et al., 2014). The hypotheses for the convergent validity analysis are that the dimensions of life satisfaction will positively correlate with positive self-concept and social skills.

Divergent validity is achieved when no correlations are obtained between the measures (Pasquali, 2007). The hypothesis is that life satisfaction will not correlate with stress symptoms, since it is described as a different construct not associated with life satisfaction, as indicated by Jovanovic and Zuljevic (2013).

Test-retest consistency, or stability, was assessed to verify the reliability of the instrument, as well as the internal consistency of the various dimensions of the MLSS-C using Cronbach’s Alpha (*α*) and composite reliability (CR).

## Methods

### Participants

The participants were 379 children (212 girls) with a mean age of 10.5 years, enrolled in one of the fifteen public schools in a city located in the state of São Paulo, Brazil. As previously reported, the sample participated in a broader study of the transition to elementary school II that occurs in Brazilian primary education between the fifth and sixth year. The main feature of this transition is a change from a classroom structure with a single teacher to a system with different teachers for different disciplines. Most children also have to move to another school. This was the case for 304 children in this study.

### Instruments

#### MLSS-C

The instrument contains 50 items, distributed among six factors: self (10 items, e.g., “I am a caring person”), compared-self (eight items, e.g., “My friends are happier than I am”), non-violence (four items, e.g., “I am angry”), family (11 items, e.g., “My family helps me when I need”), friendship (10 items, e.g., “I relate well with my colleagues”), and school (seven items, e.g., “I feel good in my school”). In each item, the response of the child indicates the degree of agreement with the statement expressed on a 5-point Likert-type response scale: (1) not at all, (5) a lot. The total Cronbach’s alpha was .93.

#### Self-Description Assessment Questionnaire 1 (SDQ1)

Designed by Marsh, Relich, and Smith (1983), the SDQ1 was adapted for Portugal by Faria and Fontaine (1990) and for the Brazilian context by Gardinal-Pizato (2010). This questionnaire is composed of 76 items covering the academic, non-academic, and overall self-concept. The academic self-concept consists of scales of self-concept in mathematics (10 items), Portuguese (10 items), and school in general (10 items). Examples of items for academic self-concept are “I really want to take math classes,” “I have good grades in Portuguese,” and “I learn very fast in every school class.” The non-academic self-concept consists of four scales: self-concept in the relationship with parents (nine items) and self-concept in relationships with friends (nine items), which are part of the social self-concept, and self-concept related to the physical appearance (nine items) and physical competence (nine items), which form the physical self-concept. Examples of items are “My parents understand me,” “I make friends easily,” “I’m ugly”, and “I like to run and play.” The instrument provides scores for the seven scales, as well as a score of general self-concept (10 items, e.g., “I do what I like to do well”). The child answers each item on a 4-point Likert-type scale: (1) totally agree, (4) totally disagree. The CFA performed for this study in Brazil provided support for the model proposed by the authors: overall self-concept: *χ* ^2^/*df.* = 2.364, Comparative Fit Index (CFI) = .92, Root Mean Square Error of Approximation (RMSEA) = .09, and the Cronbach’s alpha was .61; academic self-concept: *χ* ^2^/*df.* = 2.880, CFI = .90, RMSEA = .06, and the Cronbach’s alpha was .89; non-academic self-concept: *χ* ^2^/*df.* = 1.745, CFI = .93, RMSEA = .04, and the Cronbach’s alpha was .86.

#### Social Skills Rating System (SSRS)

Developed and validated in the USA by Gresham and Elliott (1990), the SSRS was adapted for Brazil by Del Prette and Del Prette (2003) and subsequently validated by Freitas and Del Prette (2015). It includes three evaluation questionnaires: one for children aged 6–18, one for their parents/guardians, and one for their teacher. The child self-evaluation version used in this study has 20 descriptive items of social skills. These items compose four dimensions: responsibility and cooperation (e.g., “I do my homework on time”), empathy (e.g., “I show or tell my friends I like them”), assertiveness (e.g., “I ask my classmates to enter the game”), and self-control (e.g., “I calmly end fights with my parents”). For the evaluation of the frequency of social skills in each item, three response alternatives are possible: (0) never, (1) sometimes, and (2) very often. The structure with four dimensions was confirmed by CFA in this study; *χ* ^2^/*df.* = 1.706, CFI = .90, RMR = .05, RMSEA = .04, and the total Cronbach’s alpha was .75.

#### Childhood Stress Scale (CSS)

Developed and validated by Lipp and Lucarelli (1998), this instrument aims to identify the frequency of experiencing symptoms of stress among children aged 6–14. The scale consists of 35 items answered on a Likert-type scale from 0 to 4 points. Each question is accompanied by a circle divided into four parts, and children are advised to use the crayons to color the circle to show how often what is described in each question happens. If it never happens, the circle remains uncolored (0 points); if it always happens, all four parts of the circle are colored (4 points). The items are grouped into four dimensions: physical reactions (nine items, e.g., “I have a stomach ache”), psychological reactions (nine items, e.g., “I get nervous with everything”), psychological reactions with a depressive component (nine items, e.g., “I want to disappear from life”), and psychophysiological reactions (eight items, e.g., “When I get nervous, I stutter”). In the CFA carried out with the sample of this study, the best model contained three dimensions (psychological reactions, psychological reactions with a depressive component, and psychophysiological reactions); *χ* ^2^/*df.* = 1.897, CFI = .91, RMR = .05, RMSEA = .04, and the total Cronbach’s alpha was .85.

### Data collection

After approval by the Research Ethics Committee of Faculty of Philosophy, Sciences and Letters of Ribeirão Preto and authorization from the Department of Education and Culture of the municipality, two fifth-year classes were selected from each of the 15 municipal schools. Among 823 potential participants, and out of the 1614 children enrolled in the fifth year of elementary education in 2014, parents/guardians completed the consent form to authorize the participation of 415 students. Children aged 12 or over also completed a consent form. The application of the instruments occurred collectively in two meetings in the fifth year and two meetings in the sixth year, lasting approximately an hour and a half each, on previously scheduled dates. Between the first and second collection, 39 participants were lost for three main reasons: transfers to a private school, retention in the fifth grade, and changes of city.

### Data analysis

The analysis took place on the data from the 379 children who remained in the study until the end of the data collection period. Most statistical procedures focused on data from the fifth year, with the exception of test-retest reliability, which was performed on data from the fifth and sixth grades.

The database was created with SPSS software (version 22) and AMOS software (version 18) was used for CFA. To evaluate the adjustment indices, and to compare these indices with the original 50-item model through CFA, the chi-squared test adjusted *χ* ^2^ */df.* (<3.00), the Comparative Fit Index (CFI) (>.90), the Root Mean Square Error of Approximation (RMSEA) (<.05), and Standardized Residual Root Mean Square (SRMR) (<.08) were used to evaluate the goodness of fit of the structural model (Marôco, 2010; Schweizer, 2010).

Reliability was estimated through internal consistency and stability indices. For internal consistency, Cronbach’s alpha and CR (factor loading and residual error) values higher than .70 were considered suitable indicators (Hair, Anderson, Tatham, & Black, 2009). For stability, the test-retest correlation through the intraclass correlation coefficient was used, considering the criteria from 1.00 to .75 to be high, criteria from .74 to .40 to be moderate, and criteria below .40 to be weak (Fleiss & Cohen, 1973; Silva-Junior, Vasconcelos, Griep, & Rotenberg, 2013). For the construct validity, in addition to CFA, the convergent, divergent, and discriminant (AVE) validity were evaluated.

To analyze the correlation through Pearson’s coefficient, the values set by Bryman and Cramer (2003) were used to describe correlations that were very low (≤.20), low (>.20–.40), moderate (>.40–.70), high (>.70–.90), or very high (>.90).

## Results

### CFA

All basic assumptions were fulfilled (linearity, normality, no multicollinearity, and homoscedasticity of the residual variances). From the structure proposed by Giacomoni and Hutz (2008) with six correlated dimensions (self, compared-self, non-violence, school, family, and friendship), the indices were not satisfactory (Table 1). Items 1, 4, 6, 10, 19, 21, 28, 32, 34, 36, 40, 44, 47, and 48 presented low discriminative power and low factor loading scores (below .50). Those items share some features suggesting that the children may find them difficult to understand (e.g., “I would like my friends to be different,” “I would like my family to be different”) or the fact that they describe dispositional characteristics of the child rather than their life satisfaction (e.g., “I feel calm, quiet,” “I’m smart”). After the removal of these items, the indexes improved but the value of the CFI was still lower than desired (CFI = .88). The correlation suggested by modification indices between some errors was performed: items 3 and 5 (M. I. = 46.150; “The other children are more cheerful than I am” and “My friends are more cheerful than I am”) and items 24 and 45 (M. I. = 15.093; “I like to go to school” and “I like school activities”). These correlations were justified by semantic similarities between items.

**Table 1:** Measurement model fit indexes for Multidimensional Life Satisfaction Scalefor Children for (MLSS-C)

Statistics	Model with 50 items	Model with 36 items	Model with correlations between errors
*χ* ^2^	2515.21	1138.09	1071.19
*df*	1160	579	577
*χ* ^2^/*df*	2.16	1.96	1.85
*p* value	<.01	<.01	<.01
CFI	.79	.88	.90
RMSEA	.05	.05	.04
SRMR	.07	.05	.05

*Note*: *χ* ^2^ chi-square, *df* degrees of freedom, *χ* ^2^/*df* normalized chi-square, *CFI* Comparative Fit Index, *RMSEA* Root Mean Square Error of Approximation, *SRMR* Standardized Residual Root Mean Square

**Fig. 1:**
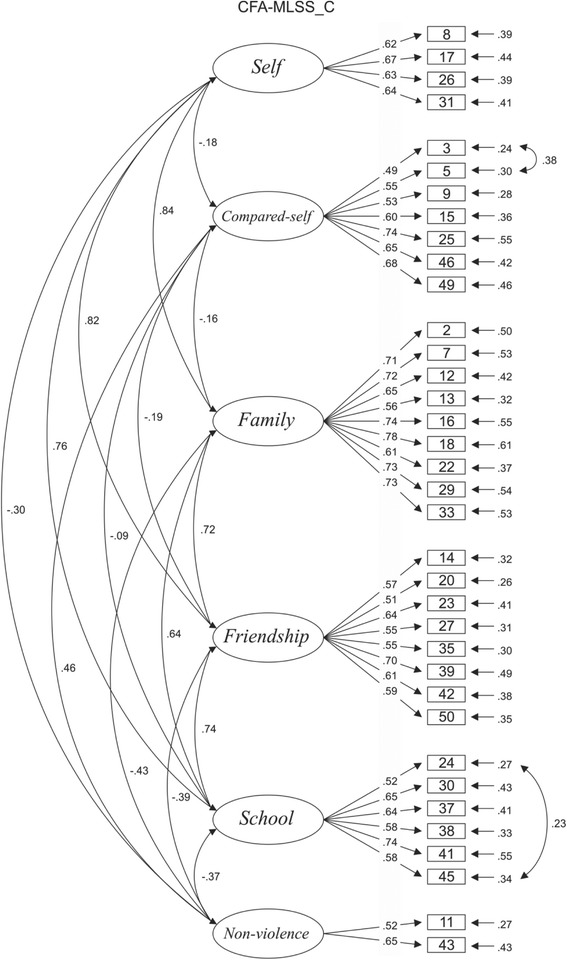
Saturation of the items and correlations between the factors obtained in the confirmatory factor analysis of the Multidimensional Life Satisfaction Scale for Children

After the introduction of these changes, a structure with six factors was obtained, showing dimensions similar to those of the original EFA (Fig. 1), with satisfactory indices of fit: (*χ* ^2^ = 1071.1, *p* < .001), *χ* ^2^/*df.* = 1.851, CFI = .90, SRMR = .05, and RMSEA = .04. The version became shorter, with 36 items, as follows: self, four items; compared-self, seven items; non-violence, two items; family, nine items; friendship, eight items; and school, six items.

### Reliability

The analysis of internal consistency provided alpha values above .70 in five of the six dimensions: self (.73), compared-self (.81), family (.88), friendship (.80), and school (.79). The non-violence dimension showed an alpha value of .50. The scale resulting from the sum of the six dimensions presented an alpha equal to .86. Accordingly, the CR values were also higher than .70 in five dimensions and lower only in the non-violence dimension (.51) (Table 2).

**Table 2:** Correlation between Multidimensional Life Satisfaction Scale for Children and the Social Skills Rating System (SSRS), the Self-Description Assessment Questionnaire (SDQ1) and the Childhood Stress Scale (CSS); square correlations between subscales (*r* ^2^); average variance extracted (AVE) and internal consistency

	Multidimensional Life Satisfaction Scale for Children^a^						
	Self (*r* ^2^)	Compared-self (*r* ^2^)	Family (*r* ^2^)	School (*r* ^2^)	Friendship (*r* ^2^)	Non-violence (*r* ^2^)	MLSS-C total
Compared-self	.150* (.022)	–	–	–	–	–	
Family	.695** (.483)	.157* (.024)	–	–	–	–	
School	.553** (.305)	.061 (.003)	.536** (.287)	–	–	–	
Friendship	.633** (.400)	.124* (.015)	.649** (.421)	.559** (.312)	–	–	
Non-violence	.149* (.022)	.279** (.077)	.235** (.055)	.220** (.048)	.215 (.046)	–	
MLSS-C total	.771**	.467**	.827**	.723**	.809**	.406**	–
SSRS total	.392**	.210**	.317**	.442**	.410**	.197**	.485**
SDQ1 total	.493**	.210**	.460**	.464**	.435**	.167**	.553**
CSS total	−.119*	−.210**	−.201**	−.081	−.101*	−.180**	−.218**
AVE	.408	.368	.481	.396	.352	.347	
CR	.73	.80	.89	.76	.81	.51	
*α*	.73	.81	.88	.79	.80	.50	

*CR* composite reliability, *α* Cronbach’s alpha

**p < .01, *p < .05

^a^ *N* = 379

The test-retest correlations were moderate in the self (.50), compared-self (.60), family (.57), school (.57), and friendship (.48) dimensions. The correlation was weak in the non-violence dimension (.38).

### Discriminant validity

Data in Table 2 indicates discriminant validity between most factors, as the values of the square correlation (*r* ^2^) are smaller than the values of the AVE. The only exception was the squared correlation between the self and family factors (*r* ^2^ = .483) which was greater than the AVE values found for the factors (.408 and .481, respectively).

### Convergent validity

According to the values defined by Bryman and Cramer (2003), the correlations (Table 2) between the SSRS (Freitas & Del Prette, 2015) and the MLSS-C were positive and moderate for the total scale (*r* = .485) and for the school (*r* = .442) and friendship (*r* = .410) dimensions. Correlations were positive and low for the self (*r* = .392), family (*r* = .317), and compared-self (*r* = .210) dimensions and very low for the non-violence (*r* = .197) dimension.

The correlation between the SDQ1 (Gardinal-Pizato, 2010) and the MLSS-C was positive and moderate (*r* = .553). In an analysis by dimension, positive and moderate relationships were found with the self (*r* = .493), family (*r* = .460), school (*r* = .464), and friendship (*r* = .435) dimensions, positive and low relationships with the compared-self (*r* = .210) dimension, and very low relationships for the non-violence (*r* = .167) dimension. These results support convergent validity.

### Divergent validity

The correlation between the CSS (Lipp & Lucarelli, 1998) and the MLSS-C was negative and low (*r* = −.218). In an analysis by dimension, no significant relationship was found with the school dimension, while negative and very low relationships were found with the self (*r* = −.119), friendship (*r* = −.101), and non-violence (*r* = −.180) dimensions and low for the family (*r* = −.201) and compared-self (*r* = −.210) dimensions. These results do not support divergent validity of the scale towards stress, except for the school dimension.

## Discussion

This study aimed to contribute to the progressive ecological validation process of the MLSS-C, initially constructed for Brazil by Giacomoni and Hutz (2008). This instrument was based on the scale developed by Huebner (1994), and the aim of the current study was to improve its accuracy and appropriateness for use with Brazilian children. CFA was used to test the theoretical structure of the instrument from the empirical data (Marôco, 2010). Through the CFA and other construct validity indicators, this study confirmed the feasibility of the model with six dimensions proposed by the authors.

The dimensions of the MLSS-C cover different positive characteristics such as self-esteem, a good mood and the ability to relate, and perceived relationships within different contexts (family, school, and peers). The information assessed through the MLSS-C also comprises information regarding perceived satisfaction with fun and support, the ability to demonstrate affection, and interpersonal relationships in different contexts. It is a self-report measure that elicits self-assessment and social comparisons (with peers), leading to the production of both global and dimensional scores.

In addition to the CFA, the convergent, divergent, and discriminant validity of this scale was investigated. Our results seem to support convergent validity, since all MLSS-C dimensions show a moderate positive correlation with self-concept and social skills. The results concerning social skills are in line with those reported for the MSLSS by Jovanovic and Zuljevic (2013) and Zappulla et al. (2014) with adolescents. Both studies showed associations between dimensions of life satisfaction and positive relationships. Concerning self-concept, there is also some previous evidence of association between life satisfaction and positive self-evaluations in children and adolescents. Giacomoni and Hutz (2008) found a positive correlation between a measure of self-esteem and the six dimensions of the MLSS-C. More recently, Zappulla et al. (2014) reported positive correlations between self-acceptance and all dimensions of the MSLSS.

The results clearly support divergent validity for the school dimension of life satisfaction in relation to stress. For self, friendship, and non-violence, the correlations with stress were very low and negative. For compared self, family, and the total MSLSS score, low negative correlations with stress were observed. These last results are consistent with the findings reported by Jovanovic and Zuljevic (2013) in their study with adolescents aged 16–19. Since we worked with younger subjects in a narrow grade school range, the pattern of results we found concerning stress deserves further attention, especially with regard to its longitudinal evolution.

The discriminant validity was mostly satisfactory (below .50 for all factors). Results concerning AVE and the correlation matrix between each factor and total score suggest that the appraisals of life satisfaction are quite differentiated by domain among children of this age. The self and family dimensions were more associated with the global score, probably because they both include items that describe affection and positive characteristics of self and because they are still less influenced by meso- and macro systems’ feedback.

The internal consistency estimates were acceptable for five of the six dimensions. The non-violence dimension, however, presented a low Cronbach’s alpha value and low CR. Possibly, the small number of items (there were only two: “I fight a lot with my friends” and “Fighting solves problems”) influenced this result. It is worth noticing that a lower Cronbach’s alpha value was also observed in the original version, which had four items, including “I like fights” and “I’m angry” (Giacomoni & Hutz, 2008). This dimension thus seems less reliable for drawing conclusions about children’s life satisfaction, probably because of social desirability and/or the lack of cognitive maturity for self-assessment.

Given the low consistence of this dimension, it can be argued that it should be dropped out of the scale. Nevertheless, the authors consider it untimely to do so at this stage of MLSS-C validation, since later studies may develop or improve it. As Giacomoni and Hutz (2008) suggested, one possible direction for this improvement would be to increase the number of the items of the subscale. The non-violence dimension needs further exploration because of its striking importance for primary and secondary prevention of social victimization.

A possible limitation of this study lies in the timing of the retesting, which occurred after an important contextual change: the transition from elementary school I to elementary school II, between the fifth and sixth year. This transition requires adaptation in the children, as it involves changes that have effects ranging from the daily routine to the composition of the peer group, when students from the same class are allocated to different schools. This situation probably increased the variation of the results in the sixth year, as the transition is associated with positive consequences for some adolescents in terms of quality of life, while for others, it can lead to negative results (Bélanger & Marcotte, 2013). The bias thereby introduced increases the probability that the stability of MLSS-C has been underestimated, since the children were experiencing changes due to the transition when responding to the scale for the second time.

Another limitation of this study concerns the composition of the sample. While representativeness was ensured in relation to the pool of the city’s public schools, the exclusive focus on public school students in their fifth year may have restricted the variability of the data, affecting the results of the correlations.

## Conclusions

The growing interest in the area of child and adolescent life satisfaction demands instruments with good psychometric properties. This study aimed to contribute to the progressive validation process of the MLSS-C. Together, the reported results broaden the evidence basis for the reliability and validity of this scale, suggesting that it is suitable for use with Brazilian schoolchildren in terms of both research and practice. Its multidimensional nature allows for obtaining global and specific scores, providing detailed information regarding the life satisfaction profile of the children. It would be important to add more waves to the research to investigate its longitudinal predictive power, as well as the relationship and/or differentiation between dimensions as children grow older and progress through school.

Considering the considerable cultural and socioeconomic diversity of Brazil, future studies should include samples from different states and cities to consolidate the ecological validity of the instrument. For further evidence of the scale’s external validity, it would also be interesting to triangulate information obtained through self-report with quantitative and qualitative information collected from parents, teachers, and other significant people in the life of the children.
